# Health insurance is associated with dental care use among university students in Washington State

**DOI:** 10.1186/s12903-023-02724-6

**Published:** 2023-01-17

**Authors:** Courtney M. Hill, Darragh Kerr, Donald L. Chi

**Affiliations:** 1 Department of Oral Health Sciences, University of Washington, 1959 NE Pacific St., Box 357475, Seattle, WA 98195-7475 USA; 2 Department of Health Systems and Population Health, University of Washington, Seattle, WA USA

**Keywords:** Oral health disparities, Health insurance, College students, Medicaid

## Abstract

**Background:**

The goal of this study was to examine the association of health insurance and preventive dental care use among university students.

**Methods:**

This secondary analysis of cross-sectional data focused on students at University of Washington in Washington state (WA) who completed a health insurance survey in 2017 (n = 3768). The exposure was health insurance (private insurance in WA [reference group], not insured, Medicaid or Medicare [public insurance], university insurance, private insurance not in WA, other) and the outcome was receiving a dental cleaning in the past 6 months. Logistic regression was used to generate odds ratios and 95% confidence intervals (CI) adjusted for confounders.

**Results:**

About 5% of university students did not have health insurance and 37% did not have a dental cleaning in the past 6 months. Compared to students with private health insurance based in WA, the odds of not receiving a dental cleaning were 3.90 times greater for university students with no health insurance (95% CI 2.74, 5.55; p < .001) and 3.08 times greater for publicly-insured university students (95% CI 2.52, 3.76; p < .001).

**Conclusions:**

University students are at risk for poor oral health behaviors. Those without health insurance and those with public insurance face barriers to preventive dental care. Efforts should be made to connect uninsured university students with insurance, dental services, and other oral health promotion activities.

**Supplementary Information:**

The online version contains supplementary material available at 10.1186/s12903-023-02724-6.

## Introduction

Oral health influences systemic health outcomes and wellbeing across the life course [1, 2]. University students are a subgroup of young adults who are particularly vulnerable to poor oral health because the transition to university is associated with lifestyle changes and increased risk factors [3]. University students are at high risk for stress [4], alcohol and drug use [5, 6], poor diet quality [7], inadequate sleep [8], and weight gain [9, 10], all of which potentially contribute to poor oral health [11–16]. Given the multiple risk factors for poor oral health for university students, finding ways to connect the most vulnerable students to oral health care could be one way to minimize the burden of oral diseases. Students who lack health insurance and who hold historically marginalized identities may be among the students most susceptible to poor oral health outcomes.

Preventive dental care, which includes dental cleanings, may be particularly relevant in protecting the oral health of university students, but no studies have examined the association in the university student subpopulation. Studies across adolescent and young adult populations have found that routine preventive dental care is associated with better self-reported oral health and fewer missing teeth and dental caries [17]. Despite the known benefits of preventive dental care, as of 2019, one-in-four U.S. university students reported they had not used preventive dental care in the past year [18]. Health insurance could be a contributing factor to preventive dental use. At least one study reported that adults without health insurance are significantly less likely to have a dental visit compared to those with health insurance, independent of dental insurance coverage [19]. Type of health insurance among university students may also be associated with non-use of preventive dental care.

Student identity may be another important determinant of dental care use. Students who hold historically marginalized identities on the basis of gender, race or ethnicity, sexual orientation, disability status, citizenship, or socioeconomic class may experience barriers accessing health care services [20] like preventive dental care and may experience downstream consequences in the form of oral diseases. Identifying which student subgroups experience the most severe inequities in access to oral health care could inform targeted multi-level interventions aimed at oral health promotion on college campuses.

The overarching goal of this study was to examine the association between health insurance and preventive dental care use among university students, a population overlooked in oral health research. We examined the association between health insurance and preventive dental care separately for undergraduate and graduate students given differences in health insurance availability. As a secondary aim, we also explored the associations between student identity (race, ethnicity, gender, sexual orientation, disability status) and preventive dental care use. We analyzed existing survey data to examine these associations. Findings are expected to guide future intervention efforts aimed at addressing oral health inequities among university students.

## Methods

### Data and study population

This was a cross-sectional study based on secondary data from a health insurance survey administered to students at a large public university (Additional files 1 and 2). An online 32-item health insurance survey that included questions on student characteristics, health insurance, and dental cleanings was administered in spring 2017. The survey was emailed to all undergraduate, graduate, and professional school students. To further ensure that the survey reached all students, administrators in each department and school were also asked to distribute the survey. In spring 2017, there were 42,335 undergraduate, graduate, and professional students enrolled at University of Washington (University of Washington Office of the Registrar 2017). The two study criteria were to have primary enrollment at the main University of Washington campus and to be actively enrolled at the time of the survey (excluding admitted/incoming students or alumni). Students who completed the survey had the option to enter a raffle for one $100 Amazon gift card, four $50 Amazon gift cards, or two $20 campus bookstore gift cards. The University of Washington Institutional Review Board granted this study exempt from ethics approval and approved a waiver for informed consent. All methods were carried out in accordance with the Declaration of Helsinki. The data are anonymous and not personally identifiable.

### Study variables

The outcome was not having a dental cleaning in the past 6 months. The main exposure was health insurance and consisted of the following categories: private insurance based in Washington state (WA) (reference group), not insured, Medicaid or Medicare (public insurance), university insurance, private insurance not based in WA, and other insurance (e.g., military insurance, international insurance, or unspecified). University insurance includes dental benefits and is only available to international students and graduate students with an eligible job appointment. During the time the survey was administered, WA Medicaid included comprehensive dental benefits for enrolled adults.

Confounders were selected a priori as variables that were conceptualized as being associated with the study exposure (health insurance) and study outcome (preventive dental care use). We included six confounders in our models: gender (man, woman, transgender, non-binary, or prefer not to say), age group (15–19 years, 20–23 years, 24–28 years, > 28 years), race or ethnicity, sexual orientation (heterosexual, LGBTQ+, unsure, other [not specified], prefer not to identify), citizenship (U.S. citizen, student visa, lawful permanent resident, undocumented, not stated) and disability status. Race or ethnicity included six categories: Asian or Asian American, Black or African American, white, Hispanic, mixed race or ethnicity, and other which included groups with low representation (Alaska Native or American Indian and Hawaiian or Pacific Islander). We also measured student classification (undergraduate, graduate or professional, other). Other students included post-baccalareature students and non-matriculated graduate students.

### Statistical analyses

We calculated descriptive statistics for the sample of university students who completed the survey (n and %). Then, we generated odds ratios (OR) with 95% confidence intervals (CI) using logistic regression to examine bivariate associations. Lastly, we used multivariable logistic regression models to estimate the associations adjusted for confounders. Based on the hypothesis that health insurance access may differ between graduate/professional and undergraduate students, we evaluated whether the association between health insurance and dental cleaning in the past 6 months was different for these two student groups using a stratified analysis. Observations with missing data were dropped for a compete-case analysis. Statistical analyses were completed using SPSS Version 25 (IBM Corp. 2017).

## Results

### Descriptive statistics

Of 42,335 eligible university students, 3887 (9.2%) completed the health insurance survey. Seventy-nine respondents were excluded because their primary enrollment was not at the main campus and 40 additional were excluded because they were not current students, which resulted in a final sample size of 3768.

The mean age of the sample was 24.4 years. Two-thirds (66.7%) of the sample were women, 23.2% were 15–19 years old, 31.5% were 20–23 years old, and 32.3% were 24 years or older (Table 1). About one-half of the students in the sample were white (53.6%), 25.8% were Asian or Asian American, and 10.6% were mixed race. Hispanic, Black or African American, and Alaska Native/American Indian or Native Hawaiian or other Pacific Islander students each constituted less than 5% of the sample. About 13.7% of the sample identified as LGBTQ + . International students, represented by those with a student visa, were 8.3% of the sample. Almost seven-percent of students reported that they had a disability. About three-fifths (56.7%) of the sample were undergraduate students and 40.0% were graduate students. About two-fifths of university students reported they did not have a dental cleaning in the past 6 months. About 5% of students had no health insurance. Among insured students, the most common source of insurance was private insurance based in WA (36.2%), followed by Medicaid or Medicare (20.1%) and university insurance (16.7%).

**Table 1:** Characteristics of university students who completed a health insurance survey in 2017 at University of Washington (N = 3768)

Characteristic	University students who completed a health insurance survey	
	n	%
Dental cleaning in past 6 months		
No	1390	36.9
Yes	2319	61.5
No response	59	1.6
Health insurance		
Not insured	181	4.8
Private insurance in WA	1365	36.2
Medicaid or Medicare	758	20.1
University	630	16.7
Private insurance not in WA	436	11.6
Other^1^	309	8.2
Does not know/no response	89	2.4
Gender		
Man	1136	30.1
Woman	2515	66.7
Transgender, non-binary, or prefer not to say	90	2.4
No response	27	0.7
Age group (years)		
15–19	874	23.2
20–23	1186	31.5
24–28	868	23.0
29–76	727	19.3
No response	113	3.0
Race or ethnicity		
Asian or Asian American	971	25.8
Black or African American	52	1.4
White	2019	53.6
Hispanic	140	3.7
Mixed race	401	10.6
Other^2^	146	3.9
No response	39	1.0
Sexual orientation		
Heterosexual	3032	80.5
LGBTQ+^3^	515	13.7
Unsure, other, prefer not to identify	192	5.1
No response	29	0.8
Citizenship		
U.S. citizen	3267	86.7
Student visa	311	8.3
Lawful permanent resident	107	2.8
Undocumented or not stated	57	1.5
No response	26	0.7
Disability		
Yes	247	6.6
No	3377	89.6
Prefer not to say	114	3.0
No response	30	0.8
Student classification		
Undergraduate	2138	56.7
Graduate or professional	1508	40.0
Other	96	2.5
No response	26	0.7

WA, Washington state

^1^Includes military insurance and international insurance

^2^Race/ethnicity categories with a small number of responses were combined (i.e. Native Hawaiian or Pacific Islander, American Indian or Alaska Native, not specified)

^3^LGBTQ + is an initialism that means Lesbian, Gay, Bisexual, Transgender, Queer or Questioning and the plus-sign signifies a number of other identities, and is included to keep the abbreviation brief when written out

### Health insurance and dental cleaning

The crude associations between health insurance and the odds of dental cleaning indicated that compared to students with private insurance in WA, students in other health insurance groups had statistically significantly higher odds of not receiving a dental cleaning in the past 6 months (Table 2). Compared to students with private health insurance based in WA, the odds of not receiving a dental cleaning in the past 6 months were 3.90 times greater for students with no health insurance after adjusting for confounders (95% CI 2.74, 5.55; p < 0.001) Publicly-insured students had 3.08 times the adjusted odds of not receiving a dental cleaning compared to university students with private insurance based in WA (95% CI 2.52, 3.76; p < 0.001).

**Table 2:** The association of health insurance and not receiving a dental cleaning in the past 6 months among university students that completed a health insurance survey in 2017

Variable	Did not have a dental cleaning in past 6 months			
	OR (95% CI)	p	AOR^1^ (95% CI)	p
Health insurance		< .001		< .001
Private insurance in WA	Ref		Ref	
Not insured	4.27 (3.09, 5.91)	< .001	3.90 (2.74, 5.55)	< .001
Medicaid or Medicare	3.08 (2.55, 3.72)	< .001	3.08 (2.52, 3.76)	< .001
University	2.40 (1.96, 2.93)	< .001	1.47 (1.14, 1.89)	< .001
Private insurance not in WA	1.48 (1.17, 1.87)	.001	1.44 (1.13, 1.84)	.02
Other^2^	2.29 (1.77, 2.97)	< .001	1.54 (1.15, 2.06)	.04
Gender	.14		.24	
Man	Ref		Ref	
Woman	0.87 (0.76, 1.01)	.07	0.93 (0.79, 1.01)	.36
Transgender; gender variant, non-conforming; or not stated	1.09 (0.70, 1.68)	.71	0.65 (0.38, 1.11)	.12
Age group (years)	< .001		.02	
15–19	Ref		Ref	
20–23	1.47 (1.22, 1.77)	< .001	1.38 (1.12, 1.69)	.002
24–28	1.63 (1.34, 1.99)	< .001	1.39 (102, 1.90)	.04
> 28	1.32 (1.07, 1.63)	.009	1.27 (0.92, 1.74)	.15
Race or ethnicity	< .001		.06	
White	Ref		Ref	
Asian or Asian American	1.50 (1.28, 1.76)	< .001	1.07 (0.88, 1.30)	.52
Black or African American	1.46 (0.84, 2.56)	.18	1.55 (0.83, 2.89)	.17
Mixed race	1.22 (0.98, 1.53)	.07	1.25 (0.98, 1.58)	.07
Hispanic or Latino	1.27 (0.89, 1.80)	.19	0.93 (0.63, 1.38)	.72
Other^3^	2.36 (1.68, 3.31)	< .001	1.66 (1.11, 2.48)	.01
Sexual orientation	.007		.51	
Heterosexual	Ref		Ref	
LGBTQ+^4^	1.18 (0.98, 1.43)	.08	1.25 (1.01, 1.55)	.04
Unsure, other, prefer not to identify	1.51(1.13, 2.03)	.006	1.34 (0.94, 1.90)	.12
Citizenship	< .001		< .001	
U.S. citizen	Ref		Ref	
Student visa	5.05 (3.89, 6.56)	< .001	5.23 (3.79, 7.20)	< .001
Lawful permanent resident	1.31 (0.88, 1.94)	.18	1.23 (0.80, 1.89)	.35
Undocumented or not stated	2.02 (1.19, 3.40)	.009	1.59 (0.84, 2.99)	.15
Disability	.03		.30	
No	Ref		Ref	
Yes	1.12 (0.85, 1.46)	.42	1.11 (0.83, 1.50)	.48
Prefer not to say	1.65 (1.13, 2.41)	.009	1.38 (0.89, 2.14)	.15
Student classification	.007		.29	
Undergraduate	Ref		Ref	
Graduate or professional	1.18 (1.03, 1.35)	.07	0.99 (0.76, 1.28)	.92
Other	3.25 (0.41, 1.04)	< .001	0.67 (0.39, 1.13)	.13

OR, odds ratio; AOR, Adjusted odds ratio; CI, confidence interval; WA, Washington state

^1^Multivariable logistic regression was based on sample with complete observations for analytical variables (n = 3497)

^2^Includes military insurance and international insurance

^3^Race/ethnicity categories with a small number of responses were combined (i.e. Native Hawaiian or Pacific Islander, American Indian or Alaska Native, not specified)

^4^LGBTQ+ is an initialism that means Lesbian, Gay, Bisexual, Transgender, Queer or Questioning and the plus-sign signifies a number of other identities, and is included to keep the abbreviation brief when written out

### Student identity and dental cleaning

In terms of student identities, age group, race or ethnicity, sexual orientation, citizenship, disability status, and student classification were all statistically significantly associated with odds of not receiving a dental cleaning in the past 6 months before adjusting for confounders. After adjusting for confounders, international students, represented by those with a student visa, had 5.23 times the odds of not having a dental cleaning in the past 6 months compared to students who reported they were U.S. citizens (95% CI 3.79, 7.20; p < 0.001). Students who identified as Alaska Native, American Indian or Native Hawaiian or other Pacific Islander had 1.66 times greater odds of not receiving a dental cleaning compared to white students (95% CI 1.11, 2.48; p = 0.01). Compared to heterosexual students, those who identified as LGBTQ + had 1.25 times greater odds of not receiving a dental cleaning (95% CI 1.01, 1.55; p = 0.01).

### Stratification by student classification

The mean age of undergraduate students was 20.1 years and the mean age of graduate and professional students was 29.0 years. Stratified analysis showed that the association between no health insurance and public insurance with receiving a dental cleaning was similar between undergraduate and graduate students (Table 3). In contrast, graduate students who had university insurance did not have statistically significantly different odds of receiving a dental cleaning compared to graduate students with private insurance in WA (OR 1.36; 95% CI 0.99, 1.87; p = 0.06). The odds of not receiving a dental cleaning were higher at older ages of undergraduate students and lower for older ages of graduate students. In addition, Black or African American graduate students had higher odds of not receiving a dental cleaning, while there was no similar association for undergraduate students.

**Table 3:** The association of health insurance and not receiving a dental cleaning in the past 6 months among university students that completed a health insurance survey in 2017, stratified by undergraduate and graduate/professional student status

Variable	Did not have a dental cleaning in past 6 months			
	Undergraduate students		Graduate/professional students	
	AOR^1^ (95% CI)	p	AOR^1^ (95% CI)	p
Health insurance		< .001		< .001
Private insurance in WA	Ref		Ref	
Not insured	3.61 (2.32, 5.61)	< .001	5.04 (2.55, 9.96)	< .001
Medicaid or Medicare	2.81 (2.15, 3.66)	< .001	3.25 (2.28, 4.64)	< .001
University	3.59 (1.65, 7.84)	.001	1.36 (0.99, 1.87)	.06
Private insurance not in WA	1.37 (1.01, 1.85)	.04	1.51 (0.95, 2.39)	.08
Other^2^	1.47 (1.02, 2.11)	.04	1.73 (0.94, 3.18)	.08
Gender	.11		.07	
Man	Ref		Ref	
Woman	1.07 (0.86, 1.33)	.57	0.75 (0.58, 0.97)	.03
Transgender; gender variant, non-conforming; or not stated	0.47 (0.22, 1.04)	.06	0.58 (0.24, 1.39)	.22
Age group (years)	.01		.05	
15–19	Ref		–	–
20–23	1.32 (1.07, 1.64)	.01	Ref	
24–28	1.63 (1.02, 2.6)	.04	0.65 (0.43, 0.98)	.04
> 28	1.67 (1.02, 2.73)	.04	0.58 (0.38, 0.9)	.01
Race or ethnicity	.25		.07	
White	Ref		Ref	
Asian or Asian American	0.89 (0.69, 1.14)	.36	1.53 (1.07, 2.19)	.02
Black or African American	1.02 (0.42, 2.45)	.97	2.54 (0.95, 6.76)	.06
Mixed race	1.22 (0.89, 1.66)	.22	1.19 (0.79, 1.79)	.41
Hispanic or Latino	0.91 (0.55, 1.51)	.71	1.35 (0.68, 2.69)	.39
Other^3^	1.63 (0.93, 2.86)	.09	1.74 (0.89, 3.42)	.11
Sexual orientation	.21		.39	
Heterosexual	Ref		Ref	
LGBTQ+^4^	1.30 (0.95, 1.76)	.10	1.2 (0.86, 1.66)	.28
Unsure, other, prefer not to identify	1.21 (0.76, 1.91)	.42	1.4 (0.72, 2.72)	.32
Citizenship	< .001		< .001	
U.S. citizen	Ref		Ref	
Student visa	7.06 (4.07, 12.24)	< .001	2.54 (1.64, 3.95)	< .001
Lawful permanent resident	1.37 (0.82, 2.29)	.22	0.83 (0.34, 2.02)	.68
Undocumented or not stated	1.76 (0.81, 3.8)	.15	0.55 (0.1, 2.99)	.49
Disability	.02		.67	
No	Ref		Ref	
Yes	1.23 (0.81, 1.85)	.006	1.04 (0.66, 1.64)	.88
Prefer not to say	2.59 (1.32, 5.08)	< .001	0.73 (0.35, 1.5)	.39

AOR, Adjusted odds ratio; CI, confidence interval; WA, Washington state

^1^Multivariable logistic regression was based on sample with complete observations for analytical variables (n = 1969 for undergraduate students and n = 1396 for graduate students)

^2^Includes military insurance and international insurance

^3^Race/ethnicity categories with a small number of responses were combined (i.e. Native Hawaiian or Pacific Islander, American Indian or Alaska Native, not specified)

^4^LGBTQ+ is an initialism that means Lesbian, Gay, Bisexual, Transgender, Queer or Questioning and the plus-sign signifies a number of other identities, and is included to keep the abbreviation brief when written out

## Discussion

In this study, we examined the association between health insurance and preventive dental care use among university students. We found that the odds of not having a dental cleaning in the past 6 months were greater among university students who did not have health insurance and publicly-insured university students compared to university students with private health insurance based in WA. There were also lower odds of receiving a dental cleaning among university students who hold historically marginalized identities. Lastly, results from a stratified analysis showed that graduate/professional students with university insurance did not have lowered odds of a dental cleaning. These findings can be used in the development of interventions on college campuses aimed at addressing oral health inequities.

University students without health insurance had almost four times higher odds of not receiving a dental cleaning compared to university students with private insurance based in WA. In addition, publicly-insured university students had about three times the odds of not receiving a dental cleaning compared to privately-insured university students. Given that health insurance is related to health care access and preventive care use [21], it is not surprising it was associated with preventive dental care use in this study. Consistent with our finding, a previous study reported that adults without health insurance, regardless of dental insurance, were significantly less likely to have a dental visit than insured counterparts [19]. Previous work has also highlighted similar inequities in preventive dental care use when comparing publicly-insured children to children with private insurance [22]. Publicly-insured adults are also more likely to report unmet dental needs compared to privately-insured adults [23]. While public insurance programs like Medicaid provide insurance for low-income adults and adults with disabilities, adult dental benefits are not mandatory, which perpetuates cost-related barriers to dental care for populations with limited financial flexibility [23–25]. However, dental benefits are covered for adults in WA Medicaid and coverage includes dental cleanings. There may be other explanations for the disparity in dental cleanings in our study. For instance, publicly-insured university students may face other barriers including lack of knowledge about benefits, inability to find a dental office that accepts public insurance, and actual or perceived insurance-based discrimination [26].

International students, students of color, and LGBTQ+ students all had higher odds of not receiving a dental cleaning compared to their counterparts. Similar inequities in preventive dental care use among international students and students of color were found in the National College Health Assessment [27]. Potential reasons behind this inequity could be that international students hold different beliefs and cultural practices around preventive dental care use or seek dental care in their home countries. Regardless, efforts should be made to connect international students with local dental care services. Studies across other age groups in the U.S. have documented race-based inequities in preventive dental care use regardless of insurance status [28, 29], which further supports the premise that inequities in preventive dental care use among university students may be rooted in factors like systemic racism and discrimination. Fewer studies have considered preventive dental care use among LGBTQ + communities, but historical discrimination and non-inclusive health care settings [30] may explain lower levels of preventive dental care use among the LGBTQ+ university students in this study. These findings suggest that in addition to uninsured students, students with historically marginalized identities may benefit most from referrals to discrimination-free and inclusive dental care. Additional studies that use measures of discrimination may be able to evaluate the extent to which disparities in preventive dental care use are explained by exposure to discrimination.

Graduate and professional students covered by university insurance in the study did not have lowered odds of receiving a dental cleaning compared to graduate and professional students with private insurance based in WA. This relationship did not hold for undergraduate students. One explanation could be the different availability of university insurance; university insurance is not available to undergraduate students who are not international students. Expanding availability of university insurance to undergraduate students may be one way to improve use of dental cleanings. However, more work is needed to understand the costs and benefits of expanding availability of university insurance, especially since university insurance is only available to graduate students who meet certain requirements for eligibility, such as holding a university job.

Multi-level interventions could help to address inequities in oral health care access on university campuses. Potential campus-based interventions include student outreach efforts that educate uninsured students on how to sign up for health insurance and interpret covered benefits. This is particularly important for publicly-insured students who had low odds of receiving a dental cleaning in this study, despite coverage for dental care services through WA Medicaid. In addition, college campuses with dental schools can partner with the university to offer lower-cost dental care options for students. Policy efforts should address inequities that stem from historical marginalization and connect international students with dental care resources. Campus health centers can also offer resources to improve oral health behaviors by offering oral hygiene products (including toothbrushes and fluoridated toothpaste) and opportunities to participate in interventions that reinforce optimal health behaviors. Such interventions could be implemented within on-campus residential housing settings, mobile phone apps, or social media sites but formative feasibility and sustainability research involving university students is needed to help refine promising intervention approaches. It is also possible that oral health among university students could be improved by developing programs to improve oral health literacy on college campuses [31] based on international work that has documented a link between oral health literacy and improved clinical oral health among college students [32].

### Limitations

There are three main study limitations. First, it is possible that university students who completed the student health insurance survey are not representative of the overall student population at the University of Washington because only about 10% of the eligible study population participated in the survey, which is an inherent risk of online-based convenience sample. However, the study sample was similar to the study population in terms of gender, race or ethnicity, citizenship, and student classification [33]. Second, the University of Washington is a 4-year urban flagship university that includes undergraduate, graduate, and professional students so the findings may not be generalizable to students at other types of colleges [27, 34]. In light of the potential limitations to the internal and external generalizability of this study, future studies that recruit representative students are warranted. Third, because we used a secondary data source, we did not have optimal study measures. For instance, a 6-month period for dental cleaning may not be as appropriate as a 12-month period for preventive dental care and we were unable to adjust for some key confounders including income, which may mean our associations are biased.

## Conclusion

In this study, we found that health insurance was significantly associated with preventive dental care use among university students. University students with no health insurance and publicly-insured students had the lowest odds of preventive dental care use. Addressing oral health inequities on college campuses will require targeted efforts to connect the most vulnerable students to care. In addition to direct referrals to dental care, multilevel interventions at the campus level and broader policy changes that support dental care access may be needed.

## Supplementary Information

**Additional file 1:** Data Dictionary.

**Additional file 2:** Publicly-Accessible Dataset.

## Data Availability

All data generated or analyzed during this study are included in this published article and its supplementary information files.

## Abbreviations

WA: Washington state
OR: Odds ratio
CI: Confidence interval
